# Supplementary material for: Qualitative Analysis and Componential Differences of Chemical Constituents in Taxilli Herba from Different Hosts by UFLC-Triple TOF-MS/MS
Source: Molecules. 2021 Oct 21;26(21):6373. doi: 10.3390/molecules26216373 (PMC8587550; doi:10.3390/molecules26216373)
Supplement: Supplementary file 1 [file molecules-26-06373-s001.zip › molecules-1381086-supplementary.pdf]

Table S1. Identification of 85 constituents in Taxilli Herba from 7 hosts by UFLC-Triple TOF-MS/MS.

No.	t_R /min	Molecular formula	MS^1(m/z)	MS^2(m/z)	Error /ppm	Compound	Hosts a	b	c	d	e	f	g	References
1	2.24	$C_6H_{12}O_6$	179.0561$[M-H]^-$	161.0457$[M-H-H_2O]^-$, 117.0207, 89.0248, 71.0158	0.05	Glucose	+	+	+	+	-	+	+	[19]
2	2.27	$C_5H_{10}N_2O_3$	145.0633$[M-H]^-$	128.0539$[M-H-NH_3]^-$, 127.0514$[M-H-H_2O]^-$,	0.70	Glutamine	+	+	+	+	+	+	+	[20]
3	2.54	$C_{11}H_{20}O_{10}$	311.0991$[M-H]^-$	233.0654$[M-H-C_2H_3O_2-CHOH]^-$,	1.93	Primeverose	+	-	+	+	+	+	+	[21]
4*	2.59	$C_{10}H_{13}N_5O_5$	282.0830$[M-H]^-$	150.0426$[M-H-Rib]^-$, 133.0153$[M-H-Rib-H_2O]^-$	4.90	Guanosine	+	+	+	+	+	+	+	[22]
5	2.63	$C_7H_{12}O_6$	191.0057$[M-H]^-$	173.0454$[M-H-H_2O]^-$, 127.0397$[M-H-H_2O-HCOOH]^-$,	0.05	Quinic acid	+	+	+	+	+	+	+	[23]
6	2.86	$C_7H_{10}O_5$	173.0472$[M-H]^-$	155.0407$[M-H_2O]^-$, 137.0246$[M-H-2H_2O]^-$,	1.10	Shikimic acid	+	-	+	+	+	+	+	[24]
7	3.12	$C_4H_6O_5$	133.0146$[M-H]^-$	115.0041$[M-H-H_2O]^-$, 71.0160$[M-H-H_2O-CO_2]^-$	2.60	Malic acid	+	-	+	-	+	-	+	[25]
8	4.96	$C_6H_8O_7$	191.0206$[M-H]^-$	173.0101$[M-H-H_2O]^-$, 154.9982$[M-H-2H_2O]^-$,	0.00	Citric acid	+	+	+	+	+	+	+	[26]
9	5.97	$C_{16}H_{22}O_{11}$	389.1082[M-H$^-$	227.0543$[M-Glc]^-$, 165.0544$[M-H-Glc-COOH]^-$	0.90	Monotropein	-	+	+	-	+	-	+	[67]
10*	7.93	$C_7H_6O_5$	169.0138$[M-H]^-$	125.0240$[M-H-CO_2]^-$, 107.0141$[M-H-CO_2-H_2O]^-$,<br>97.0341[M-H-CO2-CO]$^-$, 69.0374$[M-H-CO_2-2CO]^-$	2.60	Gallic acid	+	+	+	+	+	+	+	[27,28]
11	8.00	$C_{19}H_{16}O_4$	307.1029$[M-H]^-$	145.0508, 127.0397	2.60	Bisdemethoxycurcumin	+	+	+	+	+	+	+	[29]
12	8.86	$C_{13}H_{16}O_{10}$	331.0673$[M-H]^-$	179.0137$[M-H-G]^-$, 169.0253, 161.024$[M-H-G-H_2O]^-$	1.80	Glucogallin	+	+	+	+	+	+	+	[28]
13	9.03	$C_{13}H_{18}O_8$	301.0928$[M-H]^-$	139.0324$[M-H-Glc]^-$, 123.0089$[M-H-Glc-O]^-$	1.66	Tachioside	+	+	+	+	+	+	+	[26]
14	9.34	$C_{11}H_{20}O_9$	295.1045$[M-H]^-$	131.0934$[M-H-Glc]^-$, 113.0252$[M-H-Glc-H_2O]^-$,<br>85.0306, 71.016, 59.0162	0.30	(1S)-2(Acetyloxy)-1-(hydroxymethy)eth<br>yl-β-D-glucopyranoside	+	+	+	+	+	+	+	[30]
15	9.39	$C_{20}H_{20}O_{11}$	435.1129$[M-H]^-$	271.0448$[M-H-Glc]^-$, 313.0354, 151.0037, 125.0245	2.52	Homomangiferin	+	+	-	-	+	-	+	[31]
16	9.43	$C_{24}H_{20}O_8$	435.1124$[M-H]^-$	313.0509, 151.0025, 123.0085	0.23	Isochinomin	+	+	-	-	-	+	+	[32]
17	9.55	$C_{26}H_{22}O_{10}$	493.1183$[M-H]^-$	331.0654$[M-H-Gal]^-$	1.42	Fluorescein-β-D-galactopyranoside	+	+	-	+	-	+	+	[29]
18	10.16	$C_{23}H_{18}O_7$	405.1031$[M-H]^-$	169.0134, 71.0154	4.93	Toddacoumaquinone	+	+	-	-	+	+	-	[32]
19	10.31	$C_{13}H_{16}O_{10}$	331.1061$[M-H]^-$	169.0134$[M-H-Glc]^-$, 125.0240$[M-H-Glc-CO_2]$-	0.60	Gallic acid 3-O-β-D-<br>glucopyranoside	+	+	+	+	+	+	+	[33–35]
20	10.77	$C_{22}H_{16}O_5$	375.0927$[M-H]^-$	285.0437[M-H-Benzyl group]$^-$, 151.0030[^{1,3}A]$^-$	1.06	7-O-Benzyl Luteolin	+	-	-	-	-	-	-	[27]
21	11.12	$C_{24}H_{20}O_7$	419.1177$[M-H]^-$	271.0421, 151.0033	3.50	Artonol B	+	-	-	-	+	-	-	[36]
22	11.13	$C_{25}H_{20}O_9$	463.0869$[M-H]^-$	435.0761$[M-H-CO]^-$, 273.0364$[M-H-C_{10}H_{11}O_3]^-$,	0.00	Hydrocarpin	+	-	-	+	-	-	-	[37,38]
23	11.30	$C_{26}H_{30}O_{14}$	565.1547$[M-H]^-$	403.0473$[M-H-Glc]^-$, 241.0241$[M-H-2Glc]^-$	2.80	Mulberroside F	+	+	+	+	+	+	+	[39]
24*	11.38	$C_7H_6O_4$	153.0194$[M-H]^-$	109.0303$[M-H-CO_2]^-$, 101.0314$[M-H-CO_2-CO]^-$,	6.90	Protocatechuic acid	+	+	+	+	+	+	+	[27]

25	11.68	$C_{12}H_{14}O_9$	$301.0565[M-H]^-$	$283.0456[M-H-H_2O]^-$, $125.0241[M-H\text{-}Glc\ UA]^-$, $107.0144[M-H\text{-}Glc\ UA-H_2O]^-$	0.00	2,6-Dihydroxyphenyl-β-D-glucopyranosiduronic acid	+	+	-	+	-	+	+	[40]
26	12.24	$C_9H_8O_3$	$163.0401[M-H]^-$	$119.0482[M-H-CO_2]^-$, $93.0316[M-H-CO_2-C_2H_2]^-$	0.18	p-Coumaric acid	+	+	+	+	+	+	+	[23]
27	12.57	$C_{30}H_{26}O_{12}$	$577.1354[M-H]^-$	$451.0989[M-H-H_2O-C_6H_5O_2]^-$, $425.0835[M-H-C_8H_8O_3]^-$, $407.0733[M-H-C_8H_8O_3-H_2O]^-$,	0.40	Procyanidin B2	+	+	+	+	+	+	+	[41–43]
28	12.85	$C_{28}H_{36}O_{15}$	$611.1235[M-H]^-$	$300.9969[M-H\text{-}Neo]^-$, 275.0183	0.982	Neohesperidin dihydrochalcone	-	+	+	-	+	+	+	[29]
29	13.22	$C_{30}H_{26}O_{12}$	$577.1354[M-H]^-$	$451.0986[M-H-H_2O-C_6H_5O_2]^-$, $425.0829[M-H-C_8H_8O_3]^-$, $407.0745[M-H-C_8H_8O_3-H_2O]^-$, $289.0698[M-H\text{-}TOP]^-$, $245.0743[M-H\text{-}TOP-CO_2]^-$, $179.0721[M-H\text{-}TOP-C_6H_5O_2]$-, $125.0228[^{1,4}A]^-$	0.30	Procyanidin B1	+	+	+	+	+	+	+	[41–43]
30	13.54	$C_{45}H_{38}O_{18}$	$865.1952[M-H]^-$	$739.1671[M-H-C_6H_5O_2-H_2O]^-$, $713.1887[M-C_8H_8 0_3]^-$, $577.1301[M-H\text{-}TOP]^-$, $407.0782[M-H\text{-}TOP-C_8H_8O_3-H_2O]^-$, $289.0712[M-H\text{-}2TOP]^-$, $245.0800[M-H\text{-}2TOP-CO_2]^-$, $125.0236[^{1,4}A]^-$	3.80	Procyanidin C1	+	+	+	+	+	+	+	[41–43]
31	13.73	$C_{21}H_{32}O_{10}$	$443.1904[M-H]^-$	$425.1931[M-H-H_2O]^-$, $281.1394[M-H\text{-}Glc]^-$,	1.13	Cynaroside A	+	+	+	+	-	+	-	[44]
32*	13.96	$C_{16}H_{14}O_5$	$285.0620[M-H]^-$	$151.0184[^{1,3}A]^-$, $107.0291[^{1,3}A-CO_2]^-$	3.16	Isosakuranetin	+	-	-	-	-	-	-	[45]
33	14.15	$C_{15}H_{20}O_{10}$	$359.0967[M-H]^-$	$197.0515[M-H\text{-}Glc]^-$, $153.0028[M-H\text{-}Glc-CO_2]^-$,	1.90	Glucosyringic acid	+	+	+	+	+	+	+	[46]
34	14.32	$C_7H_6O_3$	$137.0224[M-H]^-$	$93.0334[M-H-CO_2]^-$	0.30	4-Hydroxybenzoic acid	+	+	+	+	+	+	+	[47]
35	14.34	$C_{13}H_{16}O_8$	$299.0776[M-H]^-$	$137.0241[M-H\text{-}Glc]^-$, $93.0351[M-H\text{-}Glc-CO_2]^-$	1.2	4-Hydroxybenzoic acid β-D-glucose ester	+	+	+	+	+	+	+	[48]
36*	14.74	$C_{15}H_{14}O_6$	$289.0724[M-H]^-$	$245.0235[M-H-CO_2]^-$, $179.0341[M-H\text{-}B\ ring]^-$, $167.0339[^{1,2}A]^-$, $163.0385[M-H-H_2O\text{-}B\ ring]^-$, $149.0234[^{1,3}B]^-$, $137.0237[^{1,3}A]^-$, $125.0235[^{1,4}A]^-$, $109.0289[B\ ring]^-$	0.82	(+)-catechin	+	+	+	+	+	+	+	[34,36]
37	14.75	$C_{15}H_{14}O_6$	$289.0722[M-H]^-$	$245.0300[M-H-CO_2]^-$, $179.0339[M-H\text{-}B\ ring]^-$, $167.0340[^{1,2}A]^-$, $163.0379[M-H-H_2O\text{-}B\ ring]^-$, $149.0246[^{1,3}B]^-$, $137.0237[^{1,3}A]^-$, $125.0229[^{1,4}A]^-$, $109.0199[B\ ring]^-$	0.56	Epicatechin	+	+	+	+	+	+	+	[36]
38	14.76	$C_9H_8O_4$	$179.0389[M-H]^-$	$135.0472[M-H-CO_2]^-$, $109.0440[M-H-CO_2-CO]^-$,	3.46	Caffeic acid	+	-	+	+	+	+	+	[49]

39	14.91	$C_{15}H_{20}O_9$	343.1029[M-H]$^-$	181.0490[M-H-Glc]$^-$, 135.0427[M-H-Glc-CH_4O_2]$^-$, 121.0286[M-H-Glc-COOCH]$^-$	2.04	Methyl4-(β-D-glucopyranosyloxy)-3-methoxybenzoate	+	-	+	-	+	+	-	[48]
40*	15.27	$C_{16}H_{18}O_9$	353.0875[M-H]$^-$	191.0554[M-H-caffeoyl]$^-$, 179.0365[M-H-$C_7H_{10}O_5$]$^-$	0.90	Chlorogenic acid	+	+	+	+	-	-	+	[23]
41	15.43	$C_{15}H_{14}O_7$	305.0667[M-H]$^-$	179.03325[M-H-B ring]$^-$, 125.0245[^{1,4}A]$^-$	0.10	Epigallocatechin	+	+	+	+	+	-	+	[48]
42	16.13	$C_{12}H_{22}O_8$	293.1245[M-H]$^-$	131.0710[M-H-Glc]$^-$	0.70	Ethyl3-(β-D-glucopyranosyloxy) butanoate	+	+	+	+	+	+	+	[26]
43	16.23	$C_{15}H_{12}O_7$	303.0540[M-H]$^-$	151.0051[^{1,3}A]$^-$, 152.0502[^{1,3}B]$^-$,	2.50	Taxifolin	+	+	+	+	+	+	+	[50,51]
44	16.28	$C_{21}H_{20}O_{10}$	431.1181[M-H]$^-$	269.0453[M-H-Glc]$^-$, 225.0671[M-H-Glc-CO]$^-$, 151.0033[^{1,3}A]$^-$, 107.01[^{1,3}A-CO_2]$^-$	1.47	Cosmosiin	+	-	+	-	-	-	-	[52]
45	17.59	$C_{17}H_{32}O_{10}$	395.1919[M-H]$^-$	263.0437[M-H-Xyl]$^-$, 101.0242[M-H-Xyl-Glc]$^-$	0.71	Hexyl 6-O-β-D-Xylopyranosyl-β-D-glucopyranoside	+	+	+	+	+	+	+	[53]
46	17.89	$C_{16}H_{22}O_{10}$	373.1128[M-H]$^-$	193.0511[M-H-Glc]$^-$, 149.0617[M-H-Glc-CO_2]$^-$,	0.27	Swertiamarin	+	+	+	+	+	+	+	[40]
47	18.16	$C_{37}H_{30}O_{16}$	729.1441[M-H]-	577.1324[M-H-G]$^-$, 441.0743[M-H-TOP]$^-$	1.91	Procyanidin B2 3′-O-gallate	+	+	+	+	+	+	+	[41–43]
48	18.71	$C_{15}H_{20}O_8$	327.1085[M-H]-	165.0551[M-H-Glc]$^-$, 147.0446[M-H-Glc-H_2O]$^-$,	0.92	Diyhdromelilotoside	+	+	+	+	+	+	+	[54]
49	18.97	$C_{15}H_{20}O_8$	327.1088[M-H]$^-$	165.0554[M-H-Glc]$^-$, 147.0447[M-H-Glc-H_2O]$^-$	0.9	Androsin	+	+	+	+	+	+	+	[55]
50	20.57	$C_{14}H_{18}O_9$	329.0877[M-H]$^-$	167.0338[M-H-Glc]$^-$, 123.0444[M-H-Glc-CO_2]$^-$	2.33	Phloracetophenone 4'-O- glucoside	+	+	+	-	+	+	+	[28]
51	20.96	$C_{27}H_{30}O_{14}$	577.1329[M-H]$^-$	431.0985[M-H-Rha]$^-$, 285.0244[M-H-2Rha]$^-$,	4.33	Kaempferitrin	+	+	-	-	+	-	-	[56]
52	23.64	$C_{15}H_{10}O_7$	301.1189[M-H]$^-$	283.1076[M-H-H_2O]$^-$, 271.1089[M-H-CO_2]$^-$,	0.00	Tricetin	+	+	+	+	+	+	+	[28]
53	24.42	$C_{22}H_{22}O_{12}$	477.1014[M-H]$^-$	315.0562[M-H-Glc]$^-$, 300.0131[M-H-Glc-CH_3]$^-$,	1.78	Brassicin	+	+	+	+	+	-	+	[55]
54	24.81	$C_5H_{10}O_5$	269.0647[M-H]$^-$	241.0466[M-H-CO]$^-$, 226.0396[M-H-C_2H_2O]$^-$,	0.706	Galangin	+	+	+	+	+	+	+	[57]
55	24.84	$C_5H_{10}O_5$	269.0650[M-H]$^-$	241.0507[M-H-CO]$^-$, 225.0538[M-H-CO_2]$^-$, 197.0582[M-H-CO_2-CO]$^-$, 182.0574[M-H-CO-CO_2-CH_3]$^-$	1.67	Emodin	+	+	+	+	+	+	+	[58]
56	27.32	$C_{22}H_{18}O_{10}$	441.0817[M-H]-	289.0713[M-H-G]$^-$, 271.0630[M-H-G-H_2O]$^-$, 179.0362[M-H-G-B ring]$^-$, 135.0242[^{1,3}A]$^-$, 125.0247,109.0292[B ring]$^-$	2.30	(-)-Epicatechin gallate	+	+	+	+	+	-	+	[59]
57	27.57	$C_{22}H_{24}O_{12}$	479.1198[M-H]$^-$	315.0564[M-H-Glc]$^-$, 211.0523[^{0,4}B]$^-$, 165.0558[^{1,3}B]$^-$, 151.0542[^{1,3}A]$^-$, 127.0244[B ring]$^-$	0.60	3 '-O-methyldihydroquercetin -7-O-β-D-glucoside	+	+	+	+	+	+	+	[60]

58*	28.18	$C_{28}H_{24}O_{16}$	615.0991[M-H]$^-$	463.0868[M-H-G]$^-$, 301.3056[M-H-G-Glc/Gal]$^-$	0.09	Quercetin-3-O-(6"-galloyl)-β-galactopyranside/Quercetin-3-O-(6"-galloyl)-β-glucopyranside	+	+	-	+	-	-	-	[61]
59	29.73	$C_{21}H_{20}O_{12}$	463.0866[M-H]$^-$	301.0347[M-H-Rha]$^-$, 151.0022[^{1,2}A-CO]$^-$, 107.0149[^{1,2}A-CO-CO_2]$^-$	3.40	Myricetrin	+	+	+	+	+	=	-	[62]
60	29.94	$C_{18}H_{24}O_{10}$	399.1298[M-H]$^-$	329.0508[M-H-C_5H_{10}]$^-$, 169.0150[M-H-C_5H_{10}-Glc]$^-$, 151.0072[M-H-C_5H_{10}-Glc-H_2O]$^-$, 125.0236[M-H-C_5H_{10}-Glc-CO_2]$^-$, 107.0142[M-H-C_5H_{10}-Glc-CO_2-H_2O]$^-$	0.50	Taxilluside A	+	+	+	+	+	-	+	[12]
61*	31.17	$C_{27}H_{30}O_{16}$	609.1453[M-H]$^-$	301.0354[M-H-RG]$^-$, 151.0028[M-H-RG-^{1,3}B]$^-$	1.32	Rutin	+	+	+	+	+	+	+	[63]
62*	31.30	$C_{21}H_{20}O_{12}$	463.0864[M-H]$^-$	301.0341[M-H-Gal]$^-$, 271.0233[M-H-Gal-CHO]$^-$, 151.0025[^{1,3}A]$^-$	3.40	Hyperoside	+	+	+	+	-	+	-	[62]
63	31.95	$C_{21}H_{22}O_{11}$	449.1077[M-H]$^-$	431.0945[M-H-H_2O]$^-$, 303.0478[M-H-Rha]-, 285.0380[M-H-Rha-H_2O]$^-$, 178.9977[M-H-Rha-H_2O-B ring]$^-$, 151.0030, 125.0240	0.223	Astilbin	-	+	-	-	-	-	+	[68]
64*	32.06	$C_{21}H_{18}O_{13}$	477.0655[M-H]$^-$	301.6332[M-H-Glc UA]$^-$, 283.0230[M-H-Glc UA-H_2O]$^-$, 151.0027[^{1,3}A]$^-$, 107.0140[^{0,4}A]$^-$	4.12	Quercetin 3-O-β-D-glucuronide	+	+	+	+	+	+	+	[61]
65	32.39	$C_{29}H_{36}O_{15}$	623.1976[M-H]$^-$	461.1651[M-H-$C_9H_6O_3$]$^-$, 315.1087[M-H-$C_9H_6O_3$.$C_6H_{10}O_4$]$^-$, 179.0340, 161.0232, 135.0444	0.160	Acteoside	-	+	+	+	+	+	+	[69]
66*	32.46	$C_{21}H_{20}O_{12}$	463.0872[M-H]$^-$	301.0346[M-H-Glc]$^-$, 151.0034[^{1,3}A]$^-$	2.2	Isoquercitrin	+	+	+	+	+	+	-	[64]
67*	34.39	$C_{20}H_{18}O_{11}$	433.0772[M-H]$^-$	301.0354[M-H-Ara]$^-$, 151.0037[^{1,3}A]$^-$, 107.0139[^{0,4}A]$^-$	0.12	Avicularin	+	+	+	+	+	+	+	[65]
68	34.42	$C_{21}H_{22}O_{12}$	465.1035[M-H]$^-$	313.0140[^{1,3}B]$^-$, 303.0570[M-H-Glc]$^-$, 151.0391[^{1,3}A]$^-$, 123.0085[^{1,4}A]$^-$	0.774	Taxifolin 3′-O-β-D-glucopyranoside	+	+	+	+	+	+	+	[54]
69	34.48	$C_{28}H_{32}O_{16}$	623.1975[M-H]$^-$	461.1654[M-H-Rha]$^-$, 315.1075[M-H-Rha-Glc]$^-$	0.321	Narcissin	-	-	-	+	+	+	+	[70]
70	34.99	$C_{27}H_{30}O_{15}$	593.1501[M-H]$^-$	285.0405[M-H-RG]$^-$	1.80	Kaempferol 3-rutinoside	+	+	+	+	+	+	+	[58]
71	35.11	$C_{21}H_{20}O_{11}$	447.0935[M-H]$^-$	285.0393[M-H-Glc]$^-$, 243.0497[M-H-Glc-C_2H_2O]$^-$, 241.0341[M-H-Glc-CO_2]$^-$, 151.0029[^{1,3}A]$^-$	0.50	Luteolin-7-O-glucoside	+	+	+	+	+	+	+	[13]
72	35.19	$C_{21}H_{22}O_{11}$	449.1073[M-H]$^-$	431.0945[M-H-H_2O]$^-$, 303.0478[M-H-Rha]$^-$, 285.0380[M-H-Rha-H_2O]$^-$, 178.9977[M-H-Rha-H_2O-B ring]$^-$, 151.0030,125.0240	0.891	Isoastilbin	-	-	-	-	-	-	+	[68]

73*	35.38	$C_{21}H_{20}O_{11}$	447.0921[M-H]$^-$	301.0354[M-H-Rha]$^-$, 283.0223[M-H-Rha-H_2O]$^-$, 151.0024[^{1,3}A]$^-$	2.7	Quercitrin	+	+	+	+	+	+	+	[55]
74	35.54	$C_{21}H_{22}O_{10}$	433.1131[M-H]$^-$	269.0442[M-H-Glc]$^-$, 178.9977[M-H-Glc-B ring]$^-$, 151.0028[^{1,3}A]$^-$	0.231	Isohemiphloin	-	+	-	+	+	+	+	[71]
75	36.48	$C_{22}H_{18}O_9$	425.0874[M-H]$^-$	273.0478[M-H-$C_7H_4O_4$]$^-$, 151.0390[^{1,3}A]$^-$, 137.0234, 125.0237[^{1,4}A]$^-$	0.00	(-)-Epiafzelechin 3-O-gallate	-	+	+	+	+	+	+	[72]
76	36.54	$C_{23}H_{24}O_{10}$	459.1288[M-H]$^-$	297.0762[M-H-Glc]$^-$, 191.0342[M-H-Glc-B ring]$^-$	1.74	8-methylretusin-7β-glucoside	+	+	+	+	+	+	-	[51]
77	36.71	$C_{21}H_{20}O_{11}$	447.0925[M-H]$^-$	285.0389[M-H-Glc]$^-$, 151.0022[^{1,3}A]$^-$	1.50	Astragalin	+	+	+	+	+	+	+	[65]
78	36.83	$C_{22}H_{22}O_{11}$	461.0713[M-H]$^-$	312.9958[M-H-Rha]$^-$, 285.0016[M-H-Rha-OCH_3]$^-$	0.651	Rhamnitrin	-	+	+	+	+	+	+	[73]
79	37.07	$C_{20}H_{20}O_8$	387.1141[M-H]$^-$	341.1081[M-H-CH_3-OCH_3]$^-$, 218.8840[M-H-OCH_3-2OCH_3-B ring]$^-$, 119.0350[M-H-$C_{12}H_{11}O_6$-CH_3]$^-$	1.30	5-Demethylnobiletin	+	-	-	-	-	-	-	[66]
80	37.09	$C_{21}H_{22}O_{10}$	433.1131[M-H]$^-$	311.0641[^{1,3}A]$^-$, 271.0641[M-H-Glc]$^-$, 151.0031[^{1,3}A]$^-$, 119.0511[^{1,3}B]$^-$, 107.0135[B ring]$^-$	0.02	Prunin	+	+	-	+	+	+	+	[28,55]
81	37.40	$C_{21}H_{20}O_{10}$	431.0981[M-H]$^-$	285.0406[M-H-Rha]$^-$, 227.0352[M-H-Rha-2CO]$^-$	0.60	Afzelin	+	-	+	-	-	-	-	[60,61]
82	37.89	$C_{27}H_{32}O_{14}$	579.2082[M-H]$^-$	371.1496[M-H-Glc-CH_2OH]$^-$, 256.1267[M-H-2Glc]$^-$, 228.0413[M-H-2Glc-CO]$^-$	1.33	Cascaroside A	-	-	+	+	-	+	-	[74]
83	38.64	$C_{27}H_{30}O_{14}$	577.0974[M-H]$^-$	415.0855[M-H-Rha]$^-$, 269.0745[M-H-Rha-Glc]$^-$, 225.0675[M-H-Rha-Glc-CO]$^-$	0.347	Apigenin-7-O-rutinoside	-	-	+	-	-	-	-	[75]
84	38.85	$C_{27}H_{28}O_{13}$	559.158-[M-H]$^-$	397.0178[M-H-Rha]$^-$, 280.4159[M-H-$C_{12}H_{18}O_8$]$^-$	0.10	Apimaysin	-	+	+	-	-	+	+	[76]
85*	39.51	$C_{15}H_{10}O_7$	301.0354[M-H]$^-$	273.0376[M-H-CO]$^-$, 178.9988[^{1,2}A]$^-$, 151.0030[^{1,3}A]$^-$, 121.0296[^{1,2}B]$^-$, 107.0143[^{0,4}A]$^-$	0.73	Quercetin	+	+	+	+	+	+	+	[61]

Note: (1)*:comparison with reference standards; (2) -, not detected; +, detected; (3) *Morus alba* L. (a), *Liquidambar formosana* Hance. (b), *Ilex latifolia* Thunb. (c), *Crataegus pinnatifida Bge. var. major* N.E.Br. (d), *Passiflora edulia* Sims. (e), *Pyrus pyrifolia* (Burm. F.) Nakai. (f), and *Cinnamomum camphora* (L.) Presl (g).
